# Authors’ Response to “Saving Mothers, Giving Life: Don’t Neglect the Health Systems Element”

**DOI:** 10.9745/GHSP-D-19-00313

**Published:** 2019-12-23

**Authors:** Florina Serbanescu, Claudia Morrissey Conlon, Frank Kaharuza, Masuka Musumali

**Affiliations:** a Division of Reproductive Health, Centers for Disease Control and Prevention, Atlanta, GA.; b United States Agency for International Development (USAID), Saving Mothers, Giving Life, Washington, DC.; c USAID, Kampala, Uganda.; d USAID, Lusaka, Zambia.

See related articles by Hort and Simpson and in the SMGL supplement.

On behalf of the Saving Mothers, Giving Life (SMGL) Technical Working Group, we would like to thank Holt and Simpson^1^ for their valuable insights. We also appreciate their interest in finding out more about the planning and implementation process of the initiative, the challenges encountered, and the adaptations needed to account for contextual factors in each country.

We agree with the authors on the importance of bringing attention to the valuable experiences and lessons learned during the implementation and course of the SMGL activities, including the activities directly related to health systems strengthening (HSS). We generally agree with the points raised regarding a lesser focus on the evaluation of the process of implementation of the SMGL initiative in the supplement in favor of highlighting the outcomes and impacts of the initiative on maternal and newborn health. However, we beg to differ that the implementation experiences of SGML are “not well documented.”

The articles constituting the supplement do not represent an exhaustive account of all aspects of the SMGL initiative. The select articles published in the supplement have largely focused on the outcomes of the initiative at its conclusion after 5 years of implementation. They add to already published accounts about the initial planning, implementation, and monitoring and evaluation of the SMGL interventions, including a comprehensive external evaluation of inputs and processes undertaken during the first year of the initiative.^2^ They also add to the article by Kruk and colleagues that focused on the effects of the SMGL on the health systems in Uganda and Zambia during Phase 1.^3^

In the context of describing extraordinary, effective, multisectoral, and large-scale interventions that reduced maternal mortality in the SMGL-supported districts, the supplement includes numerous examples of HSS. The articles describing the comprehensive district system strengthening approaches that led to reductions in the “Three Delays” give ample details about strategies employed at the individual, community, health facility, and district levels. Successes and challenges to implementation of these strategies and increased accountability demanded by the initiative are thoroughly documented.^4^ The SMGL model builds on an integrated approach with complex converging factors that have contributed to its success. These include a well-functioning public-private partnership, country leadership, integration into and strengthening of the existing health systems, resource mobilization, community participation, and commitments to rigorous monitoring and evaluation. The outcomes and impacts presented in the supplement were agreed-upon tracer indicators selected before the launch of the initiative and designed to capture the main effects on the maternal and child health status. The full contributions of SMGL to the health systems strengthening and the wellbeing of communities in Uganda and Zambia are not entirely amenable to quantitative monitoring and evaluation.

The fact that the authors were able to identify examples in the supplement to discuss SMGL’s successes at the macro, meso, and micro levels attests to the wealth of implementation details provided by the articles in the supplement. We echo the value of examining HSS through a more structured and formalized lens, though “there is little consensus on what health systems strengthening (HSS) entails, what the drivers of successful HSS initiatives are, and how they can be measured.”^5^ We concur that a set of well-defined and agreed-upon guiding principles and indicators, similar to those of SMGL, are very important for monitoring and evaluation of any complex health initiative or HSS.

We thank Holt and Simpson for noting the importance of HSS in global health programming and research and recognize the value of continuing to share the experiences and lessons learned during the course of planning, implementing, and evaluating the SMGL initiative. We echo their thoughts on the need for future research to tease out more firmly the critical components of HSS in Uganda and Zambia. There is a wealth of qualitative and quantitative evidence that captured these crucial experiences. Continued analyses and documentation of these aspects may include bringing forward country- and district-level insights and experiences of the SMGL initiative related to HSS. Uganda and Zambia have already embarked on a road of scaling up components of the SMGL model. Policy makers and program managers in other low- and middle-income settings where similar approaches could be used to rapidly reduce maternal mortality may greatly benefit from learning about the SMGL’s role in improving health systems.

## Notes

**First Published Online:** October 17, 2019
